# Comparative transcriptome analysis of matched primary and distant metastatic ovarian carcinoma

**DOI:** 10.1186/s12885-019-6339-0

**Published:** 2019-11-19

**Authors:** H. Sallinen, S. Janhonen, P. Pölönen, H. Niskanen, O. H. Liu, A. Kivelä, J. M. Hartikainen, M. Anttila, M. Heinäniemi, S. Ylä-Herttuala, M. U. Kaikkonen

**Affiliations:** 1 Department of Obstetrics and Gynecology, Kuopio University Hospital, Kuopio, Finland; 2 Institute of Clinical Medicine, School of Medicine, University of Eastern Finland, Kuopio, Finland; 3 Institute of Biomedicine, School of Medicine, University of Eastern Finland, Kuopio, Finland; 4 A.I. Virtanen Institute for Molecular Sciences, University of Eastern Finland, P.O. Box 1627, 70211 Kuopio, Finland; 5 Institute of Clinical Medicine, Pathology and Forensic Medicine, University of Eastern Finland, Kuopio, Finland

**Keywords:** HGSOC, Ovarian carcinoma, Metastasis, RNA sequencing, Transcriptome

## Abstract

**Background:**

High grade serous ovarian carcinoma (HGSOC) is the most common subtype of epithelial ovarian cancers (EOC) with poor prognosis. In most cases EOC is widely disseminated at the time of diagnosis. Despite the optimal cytoreductive surgery and chemotherapy most patients develop chemoresistance, and the 5-year overall survival being only 25–35%.

**Methods:**

Here we analyzed the gene expression profiles of 10 primary HGSOC tumors and 10 related omental metastases using RNA sequencing and identified 100 differentially expressed genes.

**Results:**

The differentially expressed genes were associated with decreased embryogenesis and vasculogenesis and increased cellular proliferation and organismal death. Top upstream regulators responsible for this gene signature were NR5A1, GATA4, FOXL2, TP53 and BMP7. A subset of these genes were highly expressed in the ovarian cancer among the cancer transcriptomes of The Cancer Genome Atlas. Importantly, the metastatic gene signature was suggestive of poor survival in TCGA data based on gene enrichment analysis.

**Conclusion:**

By comparing the gene expression profiles of primary HGSOC tumors and their matched metastasis, we provide evidence that a signature of 100 genes is able to separate these two sample types and potentially predict patient survival. Our study identifies functional categories of genes and transcription factors that could play important roles in promoting metastases and serve as markers for cancer prognosis.

## Background

Ovarian cancer is the seventh most common cancer in females worldwide, and the fifth most common in Europe [1]. In Europe the rate of ovarian cancer is 12.9 per 100,000 [1] whereas globally 6 per 100,000 [2]. By the time of diagnosis, nearly 70% of the patients with ovarian cancer have widely disseminated disease with intraperitoneal carcinosis and ascites. Regardless of optimal cytoreductive surgery and the high initial chemotherapy most patients with advanced stage III-IV tumours develop chemoresistance, explaining low (25–35%) 5-year overall survival [3]. EOC is classified into five maintypes: high grade serous (HGSOC), low grade serous (LGSOC), clear cell, endometrioid and mucinous carcinomas [4]. HGSOC is the most common type (70%) of EOCs and represents the poorest prognosis. LGSOC has favourable prognosis when present as small focus in borderline tumor but at advanced stages the prognosis is worse. Also mucinous tumor at stage I has excellent prognosis but when extraovarian spread is noticed the prognosis is poor [4]. Compared to HGSOC endometrioid EOC has more favourable prognosis with the 10-year OS rates 68.4% for endometrioid and 18.4% for serous histology has been reported [5]. Similar to endometrioid, also clear cell tumors are associated with endometriosis. Clear cell carcinoma is usually considered a high grade malignancy with unfavourable prognosis at advanced stages but in stage IA patients 80–90% 5-year survival is noticed [4]. Despite EOC subclassification, the standard treatments including cytoreductive surgery and platinum-based chemotherapy combined with paclitaxel remain the same for all patients. Understanding the distinct molecular characteristics of the tumors would therefore offer the possibility to develop personalized cancer treatments. Moreover, knowledge of the different molecular and genetic patterns of primary tumors compared to metastases might improve the development of targeted therapies.

Despite large number of studies profiling the transcriptome of EOC primary ovarian tumors, only limited number of reports have compared gene expression between primary tumors and their matched metastases. These studies have identified differentially expressed genes implicated in oncogenesis, metastasis, p53 signaling [6], cell adhesion, immune related pathways [7] and cellular functions related to proliferation and apoptosis [8]. However, these studies were based on microarray and did not specifically focus on the HGSOC [6–8]. Indeed, RNA-Seq offers a number of advantages compared to microarray analysis, such as broader dynamic range of RNA expression, enhanced resolution and transcriptome complexity [9].

Aim of this study was to study the differences in the gene expression profiles of histologically validated HGSOC metastases compared to primary tumors using RNA-Seq. Samples were collected during the same cytoreductive surgery before chemotherapy. To validate our results, our data was compared to TCGA database and to the known four molecular subtypes of HGSOC described by Tothill et al. and TCGA [10, 11].

## Methods

### Sample collection

Samples of primary adnexal tumor and paired omental metastases of 10 HGSOC patients were included in the study. Primary and metastatic samples were collected in the same cytoreductive surgery before chemotherapy in each patient in Kuopio University Hospital between 2004 and 2013. The patients’ ages ranged from 44 to 75 (the median 58 years). All patients were FIGO (International Federation of Gynaecology and Obstetrics) stage IIIC (*n* = 4) or IV (*n* = 6). Histologically all tumors were high grade serous ovarian carcinomas. Samples were frozen in liquid nitrogen and stored at − 80 °C until RNA preparation. For qRT-PCR analyses paired primary tumors and omental samples of six additional HGSOC patients were included. Those patients’ ages ranged from 46 to 86 (median 67 years) and FIGO stages of the patients were IIIC (*n* = 4) or IV (*n* = 2). The samples for qRT-PCR were also collected in the same cytoreductive surgery before chemotherapy like samples for RNA–seq.

### RNA-Seq

Total RNA from tissues was isolated using Trizol (Thermo Scientific) followed by DNase treatment using the Turbo DNase kit (Thermo Scientific). Ribosomal RNA was depleted using the Ribo-Zero Gold (Illumina). Libraries were prepared as previously described by Kaikkonen et al. [12]. Briefly, the RNA was base-hydrolyzed, dephosphorylated with PNK and purified using RNA Clean & Concentrator kit (Zymo). Poly(A)-tailing was followed by cDNA synthesis using complementary poly(T)-primers containing Illumina adapter sequences. Excess oligo was removed by Exonuclease I and cDNA fragments were purified using ChIP DNA Clean & Concentrator kit. The recovered cDNA was RNaseH treated and circularized (CircLigase) and amplified for 11 cycles. The final product was ran on 10% TBE gel, gel purified (190–350 bp) and cleaned-up using ChIP DNA clean & Concentrator Kit. Sequencing was performed with the HiSeq 2000 in 50 cycle single end run at EMBL Genomic Core (Heidelberg, Germany).

### qRT-PCR analysis

RNA was isolated using TRI-reagent (Thermo Scientific). One microgram of RNA was treated with DNAse I (Thermo Scientific) and converted into cDNA using RevertAid reverse transcriptase (Thermo Scientific) and random hexamers (Thermo Scientific). Analysis of mRNA levels were done using StepOnePlus Real-Time PCR System (Life technologies), TaqMan Universal PCR Mastermix (Applied Biosystems) and gene-specific Prime PCR Probe Assays (BioRad): *AMHR2* (qHsaCEP0041252), *GATA4* (qHsaCIP0028312), *MAL* (qHsaCEP0039522), *MYOCD* (qHsaCEP0058240), *NR5A1* (qHsaCIP0028304), *PPIA* (qHsaCEP0041342), *PROK1* (qHsaCEP0024916), *SFRP2* (qHsaCEP0052530), *WIPF3* (qHsaCEP0051213), *WNT5A* (qHsaCIP0028356). Relative expressions were quantified with 2^-ΔΔCT^ method [13] using *PPIA* as the reference gene.

### Data analysis

RNA-Seq was mapped using tophat allowing up to two mismatches and reporting only one alignment for each read. Poor quality reads were filtered out (minimum 97% of bp over quality cutoff 10) and tag per base value was set to 3. RefSeq expression was quantified using ‘analyzeRNA.pl’ program in HOMER [14]. Differentially expressed genes were identified using ‘getDiffExpression’ program in HOMER with edgeR [15] and batch analysis mode for analysis of paired samples (primary vs metastasis). Thresholds of FDR < 0.1, RPKM > 1 and fold change > 2 were used. Motif enrichment for FOXL2 (BYTGTTTACWTT; GSE110093), GATA4 (NBWGATAAGR; GSE35151) and NR5A1 (TTCAAGGTCA) was tested using the ‘annotatePeaks.pl’ program with ‘–nmotifs’ option. Clustering results were generated by Cluster 3.0 [16] as detailed in each figure legend. The output from clustering was viewed using Java Treeview 1.1.6r4 [17]. For gene ontology analysis, the Functional Annotation Tool of DAVID Bioinformatics Resources 6.8 [18] and Ingenuity® Pathway Analysis (IPA®, QIAGEN Redwood City, www.qiagen.com/ingenuity) was used. For IPA® upstream regulator analysis, the top transcriptional regulators and growth factors were chosen based on most significant *P*-values (*P* < 3.5E-04) and a clear predicted activation state (− 2 < activation z-score > 2).

### Data access

The experiments performed in this study are available in GEO under the accession number GSE98281.

### TCGA OV data

Survival time and status and RSEM RNA-seq data for each TCGA OV sample was obtained from firehose GDAC, doi:10.7908/C11G0KM9.

### Metastatic signature analysis using GSVA

One hundred differentially expressed genes between primary tumors and metastases, defined as metastatic signature were used in the analysis. The gene set variation analysis (GSVA) [19], available in the R/Bioconductor package GSVA 1.22.4, was used to compute a gene set enrichment score for each TCGA OV sample with the following settings: mx.diff = F, tau = 0.25, rnaseq = T. Empirical *P*-value was computed using 1000 random permutations of genes. Same amount of genes as the observed gene set was used. The observed pathway score was compared with the random permutations of a gene set size and empirical *P*-value computed as the number of higher/lower scores in the permuted set divided by the total number of permutations. Upregulated and downregulated metastasis genes were split to individual gene sets to account for directionality of gene set enrichment. Enriched samples were required to have significant enrichment of both gene sets with *P*-value < 0.001 for Kapplan Meier survival analysis.

### Kaplan-Meier survival analysis

The R package ‘survival’ was used to draw Univariate Kaplan Meier curves comparing samples with significant enrichment of metastatic signature to rest of the samples, as indicated above. The log-rank test was computed for significance evaluation between the groups. Univariate cox proportional hazard analysis was performed for TCGA data for each 100 metastatic genes and BH method was used to adjust *P*-values.

## Results

### Analysis of differentially regulated genes

**Fig. 1:**
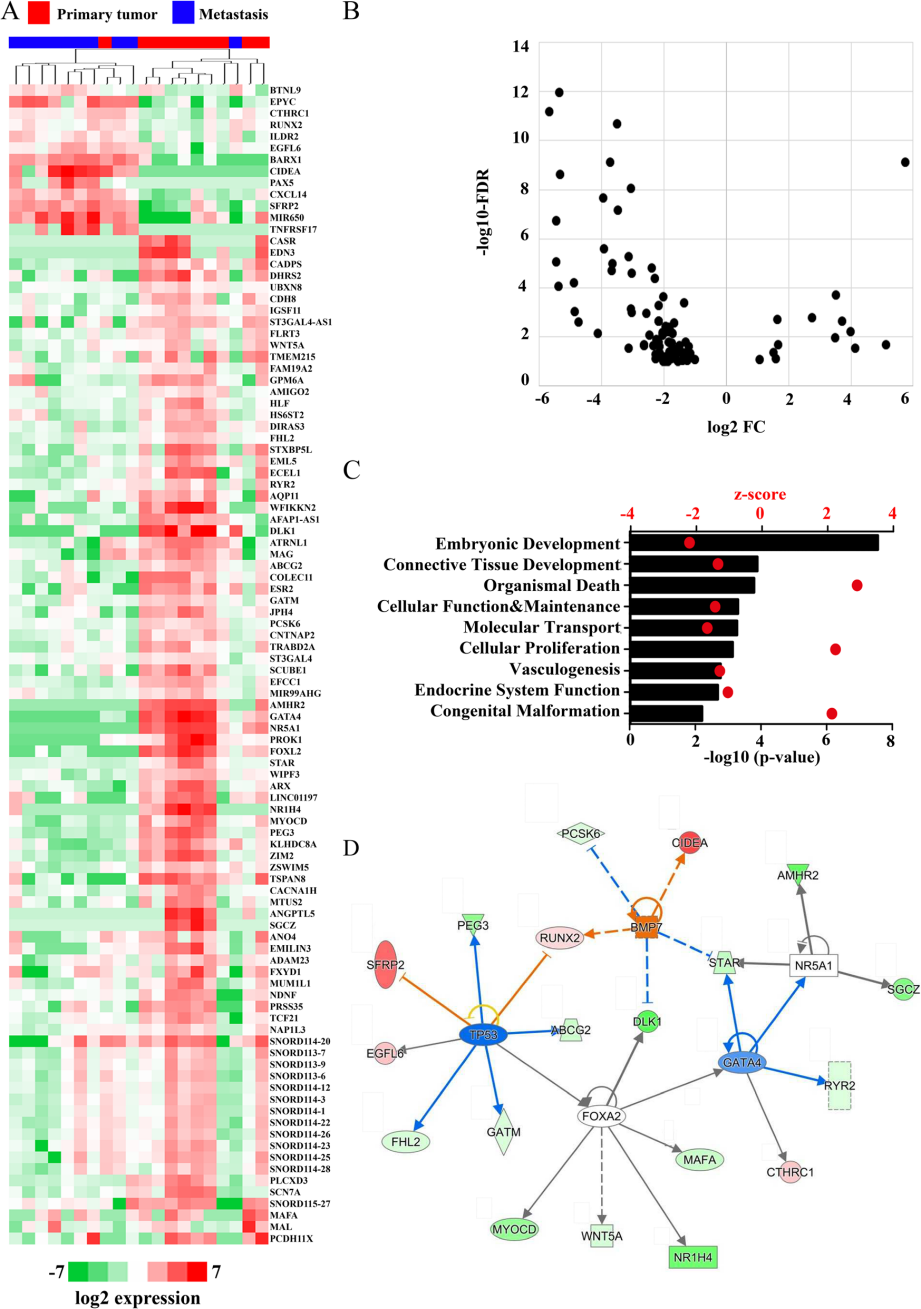
**a** Hierarchical clustering of the 100 most differentially regulated genes between primary EOC samples and their matching omental metastases based on average correlation of the log2 expression values (rpkm). Red = primary tumor, blue = metastasis. The image was generated using Java Treeview 1.1.6r4 [17] **b** Volcano plot of log2 fold change and -log10 (FDR) of the differentially regulated genes demonstrated that majority of the genes are downregulated in the omental samples. **c** IPA® gene ontology analysis of the genes demonstrated that cellular functions related to embryonic development and vasculogenesis were decreased whereas those related to organismal survival, cellular maintenance and proliferation were increased. **d** IPA® analysis of upstream transcription regulators identified activation of the *TP53* and inhibition of the *BMP7* pathways. Blue color stands for predicted inhibition and orange for predicted activation. The tones of color indicate confidence level (light = low confidence; dark = high confidence).

The gene expression profile of 10 primary tumors and 10 related metastases was analyzed using RNA-Seq. We identified 100 differentially regulated genes between the two sets, with majority (87/100) of them exhibiting downregulation in the omental samples (Fig. 1a-b: Additional file 1: Table S1). Most of the differentially regulated genes (81/100) corresponded to protein-coding accessions (NM_), whereas the remaining 19% represented non-coding RNAs (NR_), largely corresponding to small nucleolar RNAs (SNORD113–15). The gene ontology analysis (IPA) of the genes demonstrated that cellular functions related to organismal death and cellular proliferation were induced whereas those related to embryonic development, vasculogenesis, cellular function and maintenance were decreased (Fig. 1c and Additional file 2: Table S2). We further confirmed the differential mRNA expression of nine selected genes related to the top pathways, namely anti-Müllerian hormone receptor type 2 (*AMHR2),* GATA binding protein 4 *(GATA4),* myelin and lymphocyte protein *(MAL),* myocardin (*MYOCD),* nuclear receptor subfamily 5 group A member 1 *(NR5A1),* prokineticin 1 *(PROK1),* secreted frizzled related protein 2 *(SFRP2),* WAS/WASL interacting protein family member 3 *(WIPF3)* and Wnt family member 5A (*WNT5A)* using qPCR from 6 + 6 samples (Additional file 1: Fig. S1). Eight of these genes were in concordance with the RNA-Seq results suggesting high reproducibility of our results.

To study how the changes in transcriptional regulators or growth factors could explain the global changes in gene expression patterns, we performed the IPA upstream regulator analysis (Additional file 4: Table S4). The results suggested that the top upstream regulators in our data set were forkhead box protein A2 (FOXA2), receptor subfamily 5, group A, member 1 (NR5A1) and GATA-Binding Factor 4 (GATA4) (Fig. 1d). Accordingly, *NR5A1* and *GATA4* and another member of the FOXA2 family, *FOXL2*, were themselves repressed in omental samples, thus suggesting a direct role for these transcription factors in the establishment of metastasis-specific gene signature (Fig. 1a). Supporting this, 1/5 of FOXL2-targets (*MYOCD*), 4/6 GATA4-targets (*GATA4, RYR2, NR5A1, STAR*), 3/4 NR5A1-targets (*AMHR2, NR5A1, STAR*) were found to contain the respective transcription factor motif within the gene promoter (Additional file 3: Table S3). However, previous studies [20, 21] have demonstrated that majority of binding sites for FOXL2 are located outside gene promoters. In line with this, all of the predicted target genes (Fig. 1d) had FOXL2-motif located within +/− 50 kb from the transcriptional start site. In addition, tumor protein p53 (*TP53*) was found associated with a significant negative z-score (thus likely to be repressed) and bone morphogenetic factor 7 (*BMP7*) with a positive z-score (Fig. 1d). This is in line with the current knowledge where HGSOC is almost without exception accompanied with mutated *TP53* [11]. Altogether, these five upstream regulators were predicted to explain the observed gene expression changes of 22 of the differentially regulated genes (Additional file 4: Table S4).

### TCGA data comparison

**Fig. 2:**
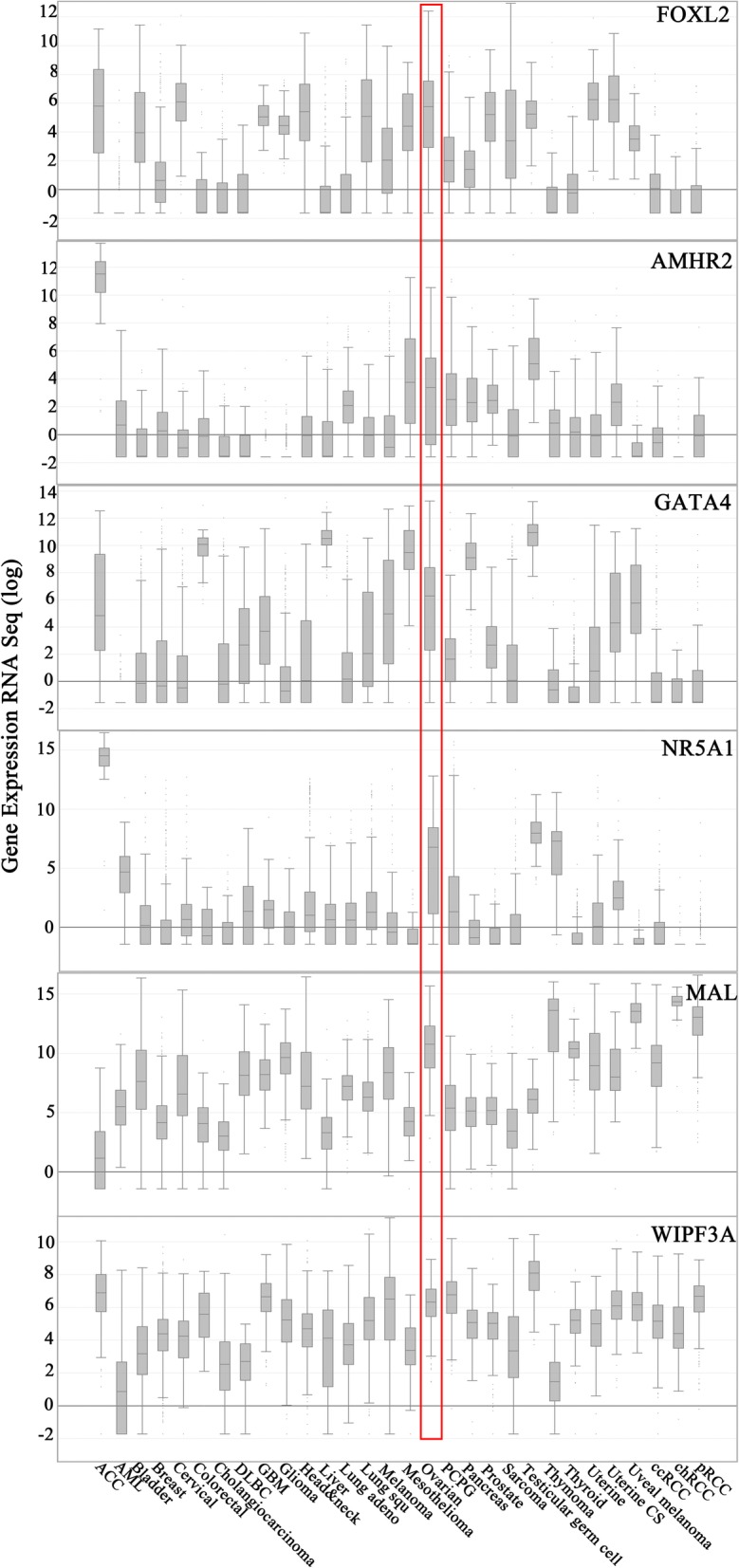
*FOXL2*, *GATA4, NR5A1, AMHR2, MAL* and *WIPF3* were found highly expressed in ovarian cancers compared to many other cancers type in TCGA dataset. The figures were downloaded from cBioPortal [43, 44]

The Cancer Genome Atlas (TCGA) contains publically available data about the genetic alterations of different cancers and also linkage to clinical features and prognosis. TCGA database contains information on the key genomic changes in over 30 different cancer types and also collection of primary ovarian tumors at the initial site of cancer, which allows comparison between different cancer types based on their gene expression profile. To see which of our differentially regulated genes were highly expressed in ovarian tumors, we compared the expression level of the 100 genes identified in the study throughout the TCGA cancer types. The analysis revealed that many of the embryonic and cell development genes are fairly high expressed in ovarian cancer including *FOXL2*, *GATA4, NR5A1, AMHR2, MAL* and *WIPF3* (Fig. 2).

**Fig. 3:**
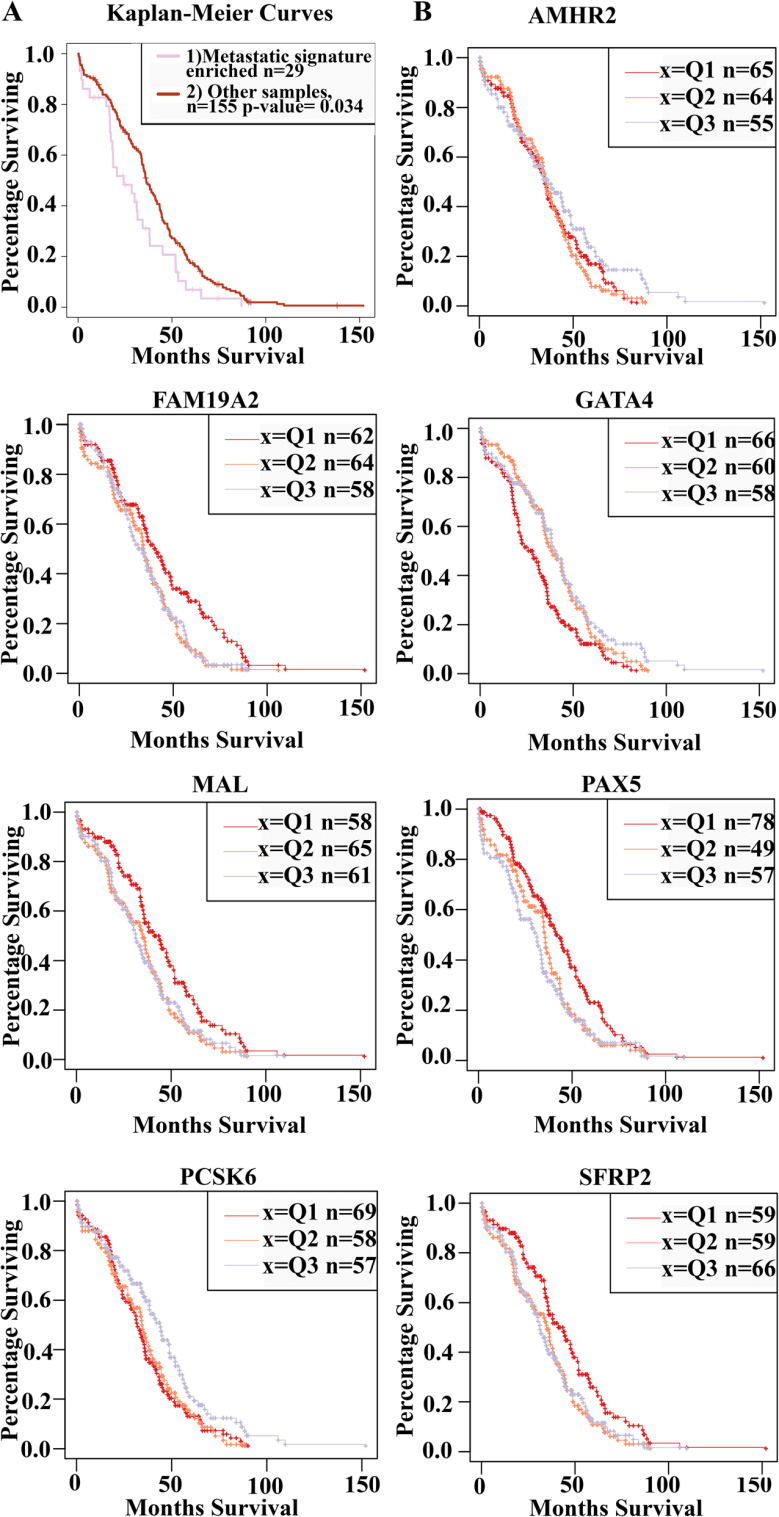
**a** Survival analysis of our differentially regulated genes in TCGA patients using GSVA tool. Gene set enrichment analysis was limited to gene sets that were upregulated for upregulated metastasis genes and downregulated for downregulated metastasis genes. 29 samples enriched with our metastasis signature showed poorer survival **b** Genes *AMHR2, FAM19A2, GATA4, MAL, PAX5, PCSK6* and *SFRP2* from univariate cox proportional hazard regression (nominal *P*-value < 0.05 Walds test) are shown as Kaplan Meier curves.

Expression in primary tumors has been associated with metastatic potential [22, 23] suggesting that metastatic gene signature could identify more aggressive tumors associated with lower survival. To this end, we conducted survival analysis based on expression profiles of TCGA primary tumors using GSVA tool. Enrichment analysis for the 100 differentially expressed genes in TCGA ovarian cancer patients thus allowed us to observe the correlation between our metastatic gene signature and survival in TCGA data. The results suggested that our metastatic gene signature could be associated with poorer survival in TCGA patients with ovarian cancer. (Fig. 3a). Among these, we were not able to identify one gene with strong predictive value but rather 7 genes that nominally affected survival (*P*-value < 0.05), including *AMHR2, GATA4, MAL, SFRP2*, Family With Sequence Similarity 19 Member A2 (*FAM19A2),* Paired Box 5 *(PAX5)* and Proprotein Convertase Subtilisin/Kexin Type 6 *(PCSK6)* (Fig. 3b; Additional file 5: Table S5). However, we acknowledge that the survival differences in TCGA samples are very small which could be due to the imperfect fit of samples for the analysis (TCGA primary tumor vs omentum). Still our results suggests that metastatic transformation of HGSOC could correlate with patient survival and identifies candidate genes that warrant future research.

### Correlation of the data to known ovarian cancer subtypes

**Fig. 4:**
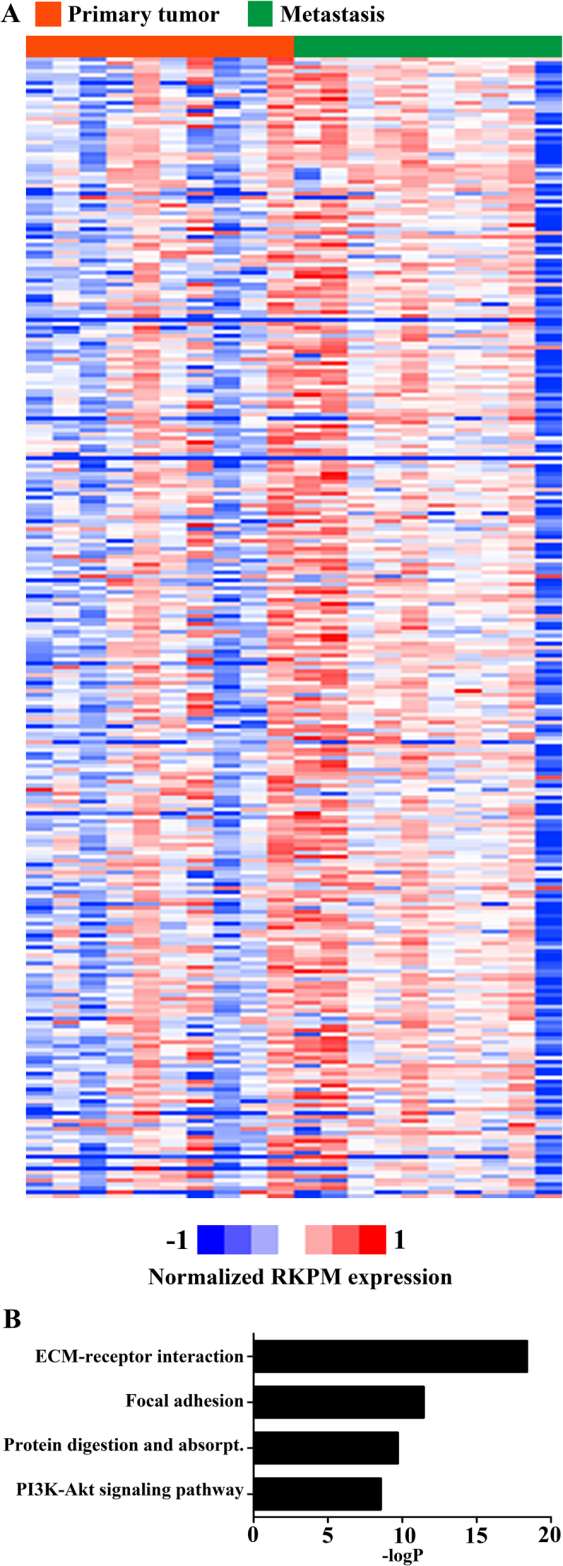
**a** Normalized and centered log2 expression values of primary tumors and metastasis of the upregulated genes of the cluster C1 [10] (blue = low expression, red = high expression, green = primary tumor, orange = metastasis) **b** The gene ontology analysis (DAVID) suggested that cellular functions related to extracellular matrix and cell cycle were activated in the genes that clustered into C1 group in Tothill et al study [10].

Several recent studies have identified molecular subtypes of ovarian cancer by gene expression profiling which aims to link expression to clinical and pathologic features. One of the most extensive study to date was performed by Tothill et al. (2008) where they conducted a whole tumor gene expression profiling of 285 predominantly high-grade and advanced-stage serous cancers of the ovary, peritoneum and fallopian tubes [10]. The authors clustered and divided the HGSOC gene expression data into four subgroups C1, C2, C4 and C5, which have been later on confirmed in the TCGA study and termed mesenchymal (C1), immunoreactive (C2), differentiated (C4) and proliferative (C5) [11]. Therefore, we next analyzed if our study samples clustered based on the ovarian cancer subtypes. Our results suggested that the upregulated genes of the cluster C1 were able to separate the primary tumor signature from omental signature (Fig. 4a) much better than any other subgroup genes (data not shown). These genes clustered into stroma signature and accordingly the gene ontology analysis (DAVID) supported the genes being implicated in functions relating to extracellular matrix and cell cycle (*ARX, CADPS, COLEC11, CTHRC1, DHRS2, DLK1, EDN3, FOXL2, GATM, GPM6A, KLHDC8A, MYOCD, PCSK6, SFRP2, TSPAN8*) (Fig. 4b). Altogether, this suggested that the mesenchymal C1 gene signature was more prominent in the omental samples compared to the primary tumors.

## Discussion

This is the first study to compare gene expression between primary EOC tumors and their matching omental metastases using RNA-seq, allowing more sensitive and deeper characterization of transcriptome compared to microarray [9]. In line with previous array-based findings, we find that the gene expression profiles of metastases differ from those of the primary tumors [6, 7]. In addition, our analysis confirms the metastasis signature being enriched for TP53 pathway and functions related to cell adhesion and proliferation [6–8]. Of the differentially expressed genes in our study, *NR1H4, CADPS, STAR, SFRP2* and *EPYC* were also observed to be differentially expressed in the similar direction in the earlier studies [6, 8]. In contrast to previous studies, our analysis identified repression of embryonic developmental genes as the biggest group of genes repressed during metastasis formation in ovarian cancer. Indeed, many of the embryonic developmental genes were also found to be highly expressed in ovarian cancer compared to many other cancers in the TCGA data, including *FOXL2, GATA4, NR5A1, AMHR2, MAL* and *WIPF3.* Of these, the first three were further identified as potential upstream regulators that could explain the observed gene expression patterns. Accordingly, the *GATA4* has been shown regulate genes involved in embryogenesis and development of the female reproductive organs, testes, GI-tract, heart and lungs [24]. Loss of this tumor suppressor gene expression has been connected to certain ovarian cancer subtypes in several studies: serous [25], clear cell [25, 26] and endometrioid [25] ovarian cancers, while mucinous ovarian cancer expresses *GATA4* [25]. This is in concordance with our finding that *GATA4* is downregulated in our metastatic gene signature in HGSOC. Statistically significant higher methylation leading to the loss of *GATA4* expression in endometrioid type compared to serous ovarian adenocarcinoma has been reported [27]. However, no correlation between *GATA4* expression and patient age, histologic type, histologic grade, stage of the disease or survival in ovarian surface epithelial carcinomas has been reported [28]. Another upstream regulator, *NR5A1* transcription factor, was also downregulated in omental samples. It encodes a human steroidogenic factor 1-protein (hSF1) that is involved in gonad development in both males and females [29]. hSF1 expression has been found to be significantly lower in ovarian cancer than in normal ovarian tissue [30] and mutations in *NR5A1* are associated with primary ovarian insufficiency [31]. The third upstream regulator identified in our analysis was *FOXA2,* that has demonstrated favorable prognosis based on TCGA data [32] and was predicted to regulate six genes that were downregulated in omental samples vs primary tumor (*DLK1, GATA4, MAFA, MYOCD, NR1H4* and *WNT5).* However, *FOXA2* was not differentially expressed in our data but rather another member of the FOX-family that encodes for transcription factor that is involved in all stages of ovarian development and function, *FOXL2* [33]. Whether FOXL2 acts to regulate predicted FOXA2-targets in ovarian cells remains to be studied. Interestingly, C134W mutation in this gene is indicated to be connected to granulosa cell tumors [34]. In a recent study *FOXL2*-positive cells were found mainly in primary and secondary ovarian tumors and very few in peritoneal seeding sites suggesting that local tissue environment could be responsible for its omental downregulation [35]. On the other hand, the changes in gene expression can also be due to changes in proportions of cell types as recently indicated by a decrease in cancer epithelial cells in ovarian cancer metastases [36]. Future studies incorporating single cell technologies are needed to evaluate the potential of the identified factors as prognostic or therapeutic targets versus cell-subtype markers.

The identification of different ovarian cancer subgroups could allow for more personalized treatments and is therefore heavily investigated. Previous molecular subtyping systems defined by TCGA and Tothill studies [10, 11] have demonstrated the existence of four molecular HGSOC subtypes represented earlier by [10] and termed them ‘mesenchymal’ (C1), ‘immunoreactive’ (C2), ‘differentiated’ (C4) and ‘proliferative’ (C5) [11]. Different molecular subgroups did not have prognostic significance in the TCGA study, but later on it was demonstrated that the proliferative and mesenchymal subtypes are associated with the poorest prognosis [37] and mesenchymal subtype with the lowest optimal-debulking rates [38]. In our study, the metastatic tumors had a gene expression signature more similar to the mesenchymal C1-group in the TCGA study compared to primary tumors. In line with this [10], the differentially expressed genes in our metastasis samples were involved in processes related to extracellular matrix signalling and cell cycle, suggesting that regulation of connective tissue deposition is upregulated in metastases. Recent study has also demonstrated that this subtype demonstrates upregulation of the TGF-β pathway [38]. Similarly, several other expression studies have reported that TGF-β pathway activities are associated with worse clinical outcomes and ovarian cancer metastasis [31, 38–40]. Therefore, tumours with the mesenchymal gene expression pattern might be considered for future trials containing TGF-β inhibitors.

Finally, survival analysis based on gene set enrichment analysis of TCGA primary tumors expression profiles revealed that the differentially regulated genes identified in this study could be indicative of poorer survival. This is in line with previous report based on 19 matched primary and omental metastatic tumors from 3 different serous adenocarcinoma types [8]. In contrast, another study showed that many good prognosis genes were more highly expressed and poor prognosis genes lower expressed in the peritoneal metastasis vs primary tumor, indicative of the metastatic lesions remaining closer to normal tissue [7]. This is in line with the expression patters of *MAL* and *FAM19A2* in our analysis. However, among the five other genes with prognostic value, genes associated with better prognosis were downregulated (*GATA4, AMHR2* and *PCSK6*) and genes with poorer prognosis were upregulated (*PAX5* and *SFRP2*) in the metastatic samples. This could reflect subtype differences of the EOCs, as patients in our study were limited to HGSOCs only. Recent reports have also identified markers related to recurrence in ovarian cancer primary tumors. These further identified networks related to TP53 and TGF-β signaling, cell cycle, leukocyte migration and cellular adhesion [41, 42]. Evidently, deciphering the molecular mechanisms and similarities of metastatic transformation and recurrence of primary tumors will be important for understanding the pathogenesis of the disease and to improve the treatment, especially in advanced stage. Despite the exploratory nature of our study, limited by low sample amounts and overall small effect on survival, our study provides many candidates that warrant future research and replication in other independent cohorts. Overall, our analysis reveals novel aspects of metastatic transformation of HGSOC, with potentially important implications for prognosis and therapy.

## Conclusions

In this study we provide evidence that the gene expression profile of primary HGSOC tumors differs from their matched metastases, and that the 100 differentially expressed genes identified could nominally predict patient survival. Identified functional categories of genes and transcription factors could play important roles in promoting metastases and serve as markers for cancer prognosis. These findings serve candidates for future research and could lead to improved treatments for HGSOC in the future.

## Supplementary information

**Additional file 1: Figure S1.** Comparison of the expression of 9 differentially regulated genes from additional 6 patients by qPCR (white bars) was in line with the RNA-Seq results (black bars).

**Additional file 1: Table S1.** The differentially expressed genes identified in our analysis.

**Additional file 2: Table S2.** Gene Ontology Analysis (Diseases or Functions Annotation) of differentially expressed genes performed using Ingenuity Pathway Analysis.

**Additional file 3: Table S3.** Number of upstream transcription factor motifs predicted within (+/− 1.5 kb of TSS) or around (+/− 50 kb from TSS) promoters of IPA predicted target genes.

**Additional file 4: Table S4.** Upstream regulators identified in our data using Ingenuity Pathway Analysis.

**Additional file 5: Table S5.** Univariate cox proportional hazard analysis for our 100 metastatic genes performed from TCGA data.

## Data Availability

All data generated and analyzed during this study are available in Gene Expression Omnibus under the accession number GSE98281.

## Abbreviations

EOC: Epithelial ovarian carcinoma
HGSOC: High-grade serous ovarian carcinoma
LGSOC: Low-grade serous ovarian carcinoma
