# Supplementary figures and images for: Comparative transcriptome analysis of matched primary and distant metastatic ovarian carcinoma
Source: BMC Cancer. 2019 Nov 19;19:1121. doi: 10.1186/s12885-019-6339-0 (PMC6862850; doi:10.1186/s12885-019-6339-0)

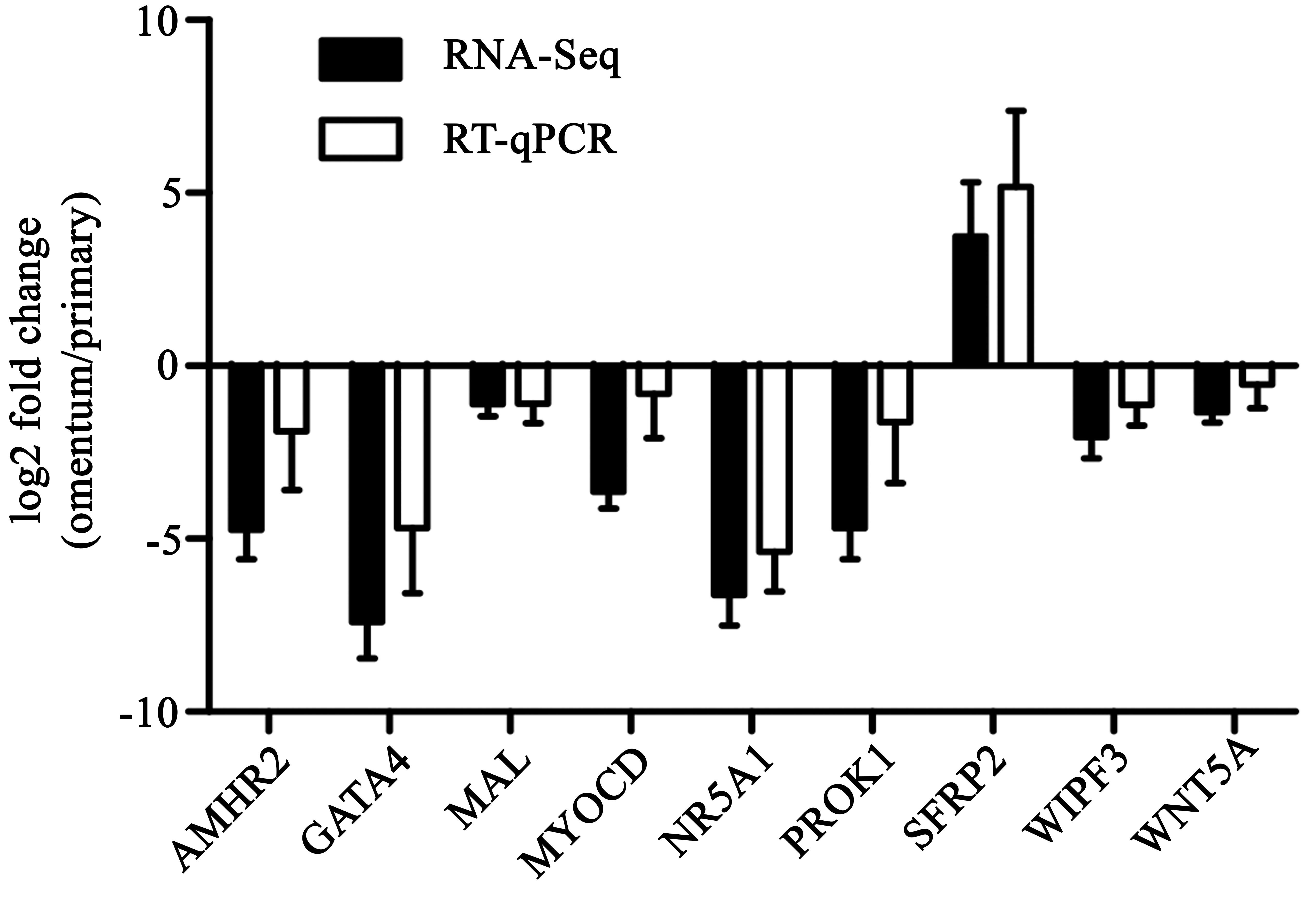

Supplement: Supplementary file 1 — Additional file 1: Figure S1. Comparison of the expression of 9 differentially regulated genes from additional 6 patients by qPCR (white bars) was in line with the RNA-Seq results (black bars). [file 12885_2019_6339_MOESM1_ESM.jpg]
